# Supplementary figures and images for: Seasonality and alternative floral resources affect reproductive success of the alfalfa leafcutting bee, Megachile rotundata
Source: PeerJ. 2024 Aug 16;12:e17902. doi: 10.7717/peerj.17902 (PMC11332388; doi:10.7717/peerj.17902)

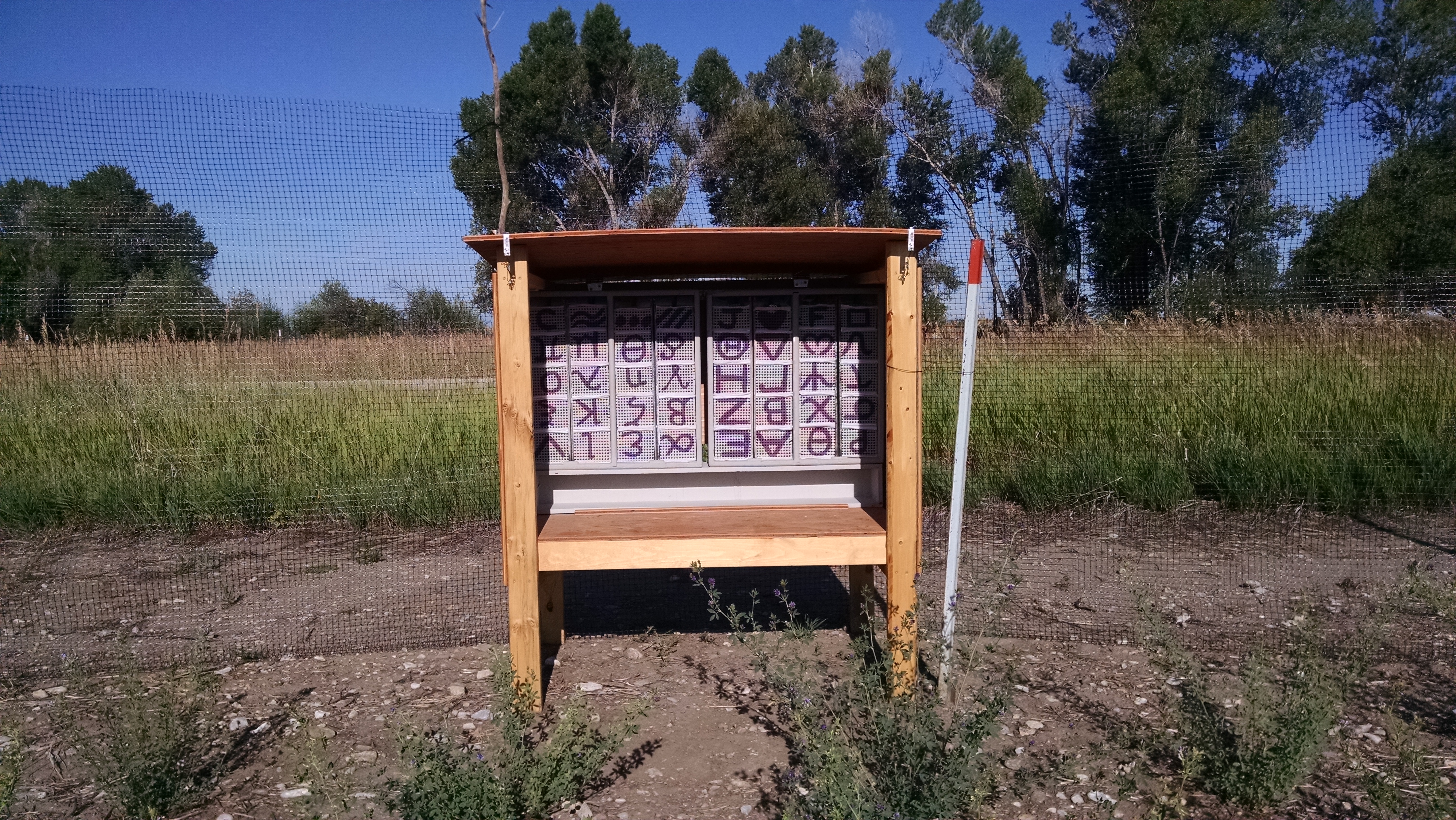

Supplement: Supplemental Information 1 — We constructed six wood nesting shelters measuring 1.4-m tall x 1.2-m wide x 0.78-m deep following O’Neill (2004). In June, we placed one bee shelter alongside the edge of each alfalfa plot and anchored the shelters to metal T-posts. Forty sterilized wood-laminate nesting blocks were placed in each shelter. Nesting blocks were taped together and backed with fabric to prevent parasites from entering the backs of the nests. Nesting tunnels measured 5.5 mm in diameter and 10-cm deep. In total, each shelter contained 4,888 tunnels. Following standard ALCB management practices, we painted the faces of the nesting blocks with symbols to provide orientation cues for nesting females (Guédot, Bosch & Kemp, 2005). Photo credit: Casey M. Delphia. [file peerj-12-17902-s001.jpg]

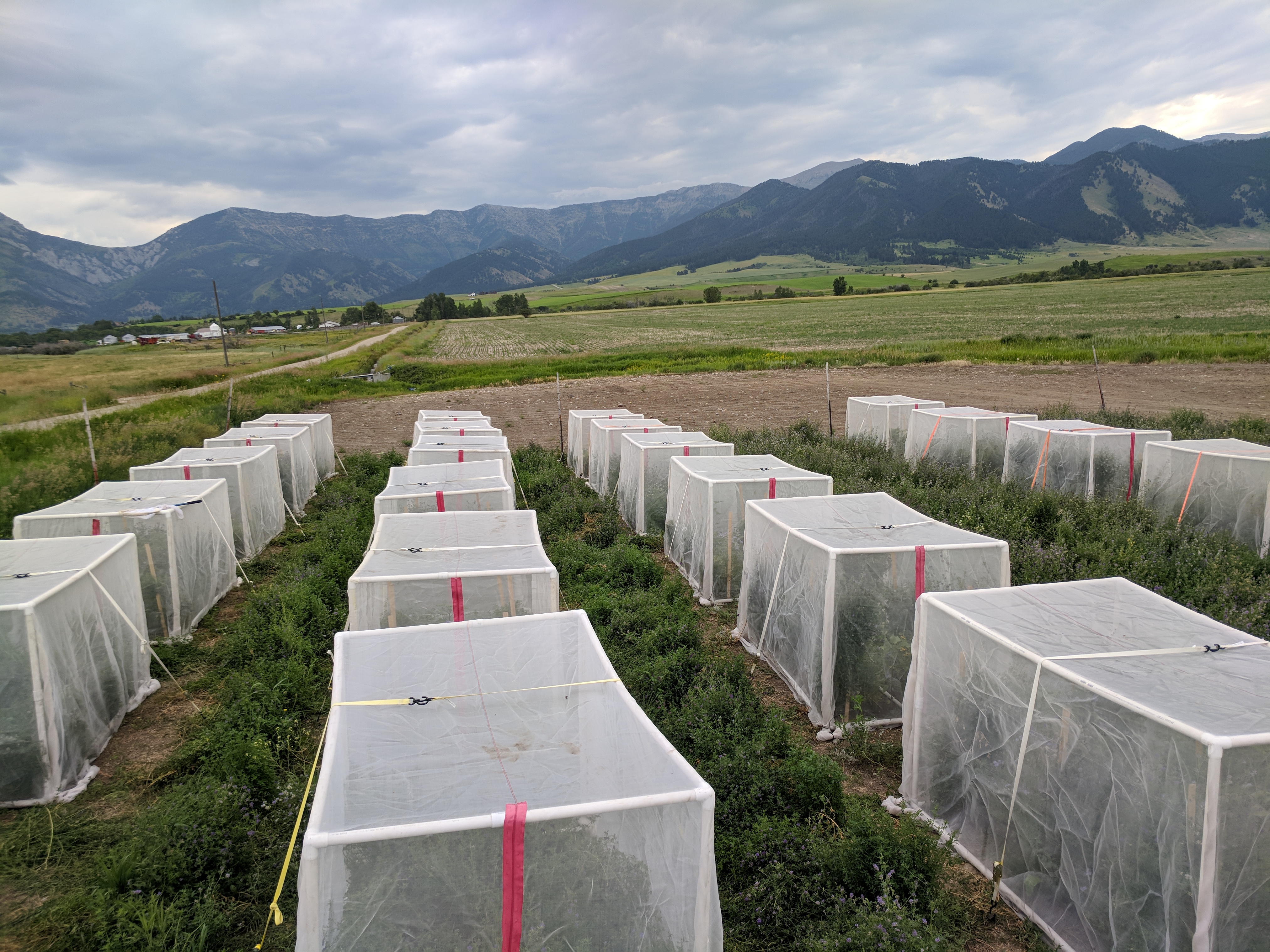

Supplement: Supplemental Information 3 — Each of 24 plots measuring 3.6 m2 was randomly assigned to one of three food resource treatments: alfalfa only, alfalfa plus wildflowers, or wildflowers only. For logistical reasons associated with irrigation, treatments were laid out such that there were four rows of six plots with two replicates of each treatment randomly assigned to a plot within each row (i.e., randomized complete block design with four blocks and three treatments with two replicates randomized within each block, N = 8 plots per treatment). Photo credit: Casey M. Delphia. [file peerj-12-17902-s003.jpg]

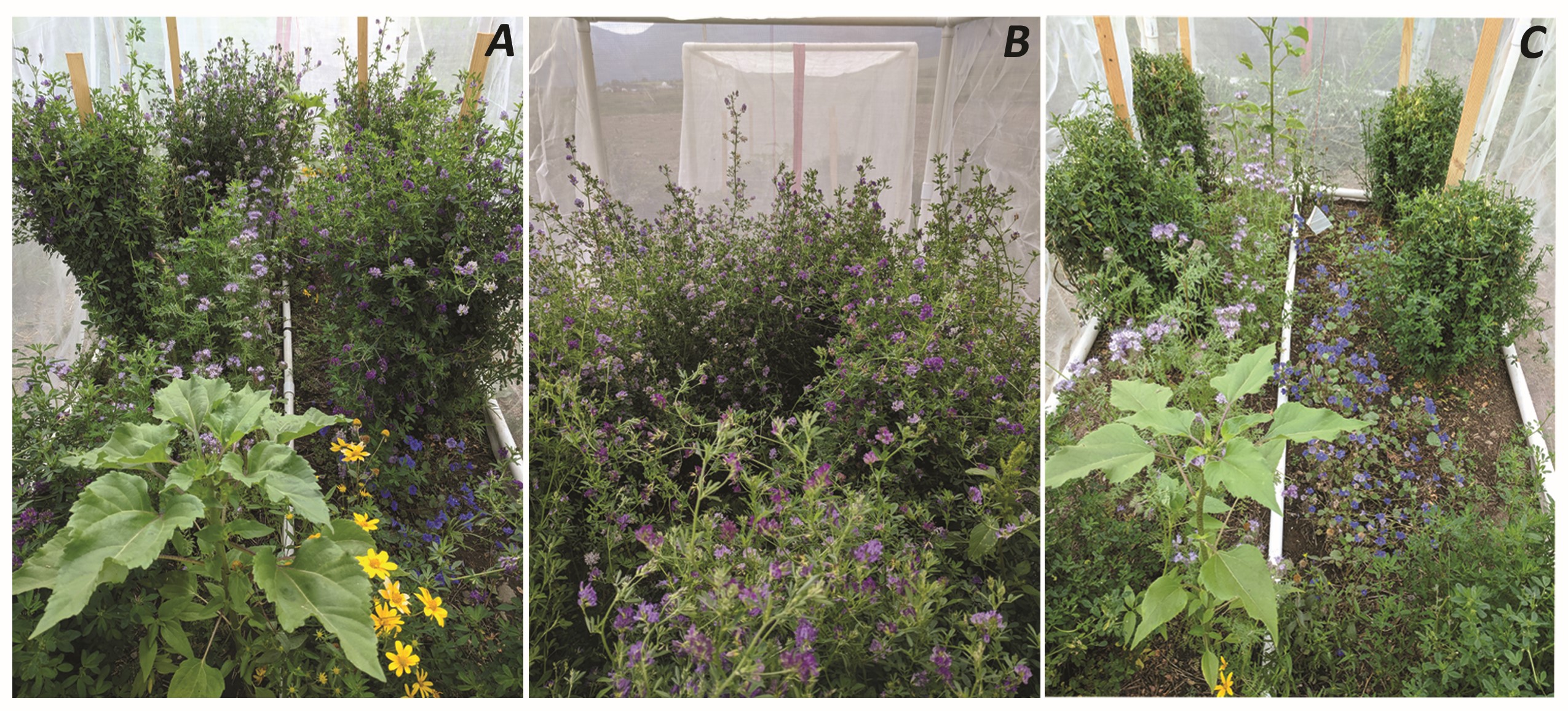

Supplement: Supplemental Information 4 — The three food resource treatments used in the cage study included: (A) alfalfa-plus-wildflowers, (B) alfalfa-only, or (C) wildflowers-only. Photo credit: Casey M. Delphia. [file peerj-12-17902-s004.jpg]
