# Supplementary material for: Seasonality and alternative floral resources affect reproductive success of the alfalfa leafcutting bee, Megachile rotundata
Source: PeerJ. 2024 Aug 16;12:e17902. doi: 10.7717/peerj.17902 (PMC11332388; doi:10.7717/peerj.17902)
Supplement: Supplemental Information 6 — ANCOVA results of the influence of wildflower treatment, week, alfalfa floral density, and shelter orientation on total lipid mass (µg) of adult female offspring that emerged in 2018 from cells provisioned over 8 weeks in 2017. P-values in boldface are significant at α = 0.05. [file peerj-12-17902-s006.docx]

|  |  | Lipid mass (µg) | |
| --- | --- | --- | --- |
| Source | df | F | P-value |
| Treatment | 1, 751 | 0.55 | 0.4581 |
| Week | 7, 751 | 10.19 | **<0.0001** |
| Shelter orientation | 1, 751 | 3.20 | 0.0739 |
| Sqrt (Alfalfa floral density) | 1, 751 | 7.94 | **0.0050** |
