# Supplementary material for: Seasonality and alternative floral resources affect reproductive success of the alfalfa leafcutting bee, Megachile rotundata
Source: PeerJ. 2024 Aug 16;12:e17902. doi: 10.7717/peerj.17902 (PMC11332388; doi:10.7717/peerj.17902)
Supplement: Supplemental Information 7 — ANOVA model results of the effect of treatment on mean bee cells with offspring (i.e., an egg), mean proportion adult emergence success, mean number females, mean number males, and mean male: female sex ratios per nest (female per-capita metric of biological relevance). P-values in boldface are significant at α = 0.05. [file peerj-12-17902-s007.docx]

|  |  | **Bee cells** | | **Emergence success** | | **Females** | | **Males** | | **Sex ratios** | |
| --- | --- | --- | --- | --- | --- | --- | --- | --- | --- | --- | --- |
| Source | df | F | P-value | F | P-value | F | P-value | F | P-value | F | P-value |
| Treatment | 2, 21 | 3.96 | **0.0348** | 8.37 | **0.0021** | 4.42 | **0.0249** | 2.57 | 0.1005 | 3.28 | 0.0574 |
