# Supplementary material for: Seasonality and alternative floral resources affect reproductive success of the alfalfa leafcutting bee, Megachile rotundata
Source: PeerJ. 2024 Aug 16;12:e17902. doi: 10.7717/peerj.17902 (PMC11332388; doi:10.7717/peerj.17902)
Supplement: Supplemental Information 8 — Repeated-measures MANOVA results of the influence of cage treatment on mean proportion of offspring per nest that died at each of five stages (i.e., egg, larva, prepupa, pupa, and unemerged adult). P-values in boldface are significant at α = 0.05. [file peerj-12-17902-s008.docx]

| Source | df | F | P-value |
| --- | --- | --- | --- |
| Treatment | 2, 21 | 0.03 | 0.9683 |
