# Supplementary material for: Seasonality and alternative floral resources affect reproductive success of the alfalfa leafcutting bee, Megachile rotundata
Source: PeerJ. 2024 Aug 16;12:e17902. doi: 10.7717/peerj.17902 (PMC11332388; doi:10.7717/peerj.17902)
Supplement: Supplemental Information 9 — Linear mixed-effects model results of the influence of cage treatment on total lipid mass (µg) of adult female offspring that emerged in 2019 from cells provisioned in 2018. Cage and nest (nested within cage) are included as random effects. P-values in boldface are significant at α = 0.05. [file peerj-12-17902-s009.docx]

| Source | df | F | P-value |
| --- | --- | --- | --- |
| **Lipid mass (µg)** |  |  |  |
| Treatment | 2, 19.96 | 8.73 | **0.0019** |
