# Supplementary material for: Seasonality and alternative floral resources affect reproductive success of the alfalfa leafcutting bee, Megachile rotundata
Source: PeerJ. 2024 Aug 16;12:e17902. doi: 10.7717/peerj.17902 (PMC11332388; doi:10.7717/peerj.17902)
Supplement: Supplemental Information 10 — Linear mixed-effects model results of the influence of pollen protein content on proportion body lipids and total lipid mass (µg) of adult female offspring that emerged in 2019 from cells provisioned in 2018 in the alfalfa-plus-wildflower treatment only. Cage and nest (nested within cage) are included as random effects. P-values in boldface are significant at α = 0.05. [file peerj-12-17902-s010.docx]

| Source | df | F | P |
| --- | --- | --- | --- |
| **Proportion body lipids** |  |  |  |
| Pollen protein content | 1, 96.23 | 0.04 | 0.8329 |
| **Lipid mass (µg)** |  |  |  |
| Pollen protein content | 1, 117.2 | 0.79 | 0.3747 |
