# Supplementary material for: Seasonality and alternative floral resources affect reproductive success of the alfalfa leafcutting bee, Megachile rotundata
Source: PeerJ. 2024 Aug 16;12:e17902. doi: 10.7717/peerj.17902 (PMC11332388; doi:10.7717/peerj.17902)
Supplement: Supplemental Information 11 — Mixed-effect linear model results of the influence of wildflower treatment and alfalfa plant biomass on alfalfa seed yields (total seed mass) in 2017. Site was included as a random effect. P-values in boldface are significant at α = 0.05. [file peerj-12-17902-s011.docx]

| Source | df | F | P-value |
| --- | --- | --- | --- |
| Treatment | 1, 51 | 1.65 | 0.2681 |
| Sqrt (Plant biomass) | 1, 51 | 22.23 | **< 0.0001** |
