# Supplementary material for: Seasonality and alternative floral resources affect reproductive success of the alfalfa leafcutting bee, Megachile rotundata
Source: PeerJ. 2024 Aug 16;12:e17902. doi: 10.7717/peerj.17902 (PMC11332388; doi:10.7717/peerj.17902)
Supplement: Supplemental Information 12 [file peerj-12-17902-s012.docx]

**Methods**

**Statistical analyses: Field study.**

***Offspring condition.*** We performed a separate univariate ANCOVA testing the effect of wildflower treatment on total lipid mass with treatment and week (ordinal) as main effects and alfalfa floral density and bee shelter orientation as covariates.

**Results**

**Field study:** Do late-season supplemental wildflower resources enhance ALCB reproduction and offspring condition?

***Offspring condition.*** Wildflower treatment did not affect total lipid mass of adult female offspring that emerged in 2018 (Table S1). However, female offspring from cells completed earlier in the nesting season had greater total lipid mass (Fig. S2). Alfalfa floral resources were negatively associated with lipid mass. Shelter orientation was not associated with lipid mass.
