# Supplementary material for: Seasonality and alternative floral resources affect reproductive success of the alfalfa leafcutting bee, Megachile rotundata
Source: PeerJ. 2024 Aug 16;12:e17902. doi: 10.7717/peerj.17902 (PMC11332388; doi:10.7717/peerj.17902)
Supplement: Supplemental Information 13 [file peerj-12-17902-s013.docx]

**Results**

**Cage study:** Do alternative wildflower resources enhance ALCB reproduction and offspring condition?

***Offspring production and survival among cages.*** Relative to the other treatments, females in the A+WF treatment provisioned more bee cells with an egg per nest (Table S2). The proportion of adult offspring per nest from A-only was higher than that from WF-only, but neither was different from A+WF (Table S2).

More female and male offspring emerged per nest from A+WF compared to WF-only, and neither was different from the A-only (Table S2). There was no difference among treatments in the number of males that emerged per nest (Table S2).

There was a marginally significant treatment effect on the mean male: female sex ratio per nest (Table S2). The mean sex ratio per nest was 2.95 ± 0.27 in the A-only treatment, 2.02 ± 0.34 in the A+WF treatment and 1.70 ± 0.45 in the WF-only treatment.

Offspring mortality per nest was similar across treatments (Table S3). Most offspring died at the larval stage (mean proportion all treatments = 0.35 ± 0.03, N = 24); followed by prepupal, pupal, unemerged adult, and egg stages).
